# Quantifying the role of motor imagery in brain-machine interfaces

**DOI:** 10.1038/srep24076

**Published:** 2016-04-07

**Authors:** Silvia Marchesotti, Michela Bassolino, Andrea Serino, Hannes Bleuler, Olaf Blanke

**Affiliations:** 1 Laboratory of Cognitive Neuroscience, École Polytechnique Fédérale de Lausanne (EPFL), Lausanne, Switzerland; 2 Center for Neuroprosthetics, EPFL, Lausanne, Switzerland; 3 Laboratory of Robotic Systems, EPFL, Lausanne, Switzerland; 4 Department of Neurology, University Hospital, Geneva, Switzerland

## Abstract

Despite technical advances in brain machine interfaces (BMI), for as-yet unknown reasons the ability to control a BMI remains limited to a subset of users. We investigate whether individual differences in BMI control based on motor imagery (MI) are related to differences in MI ability. We assessed whether differences in kinesthetic and visual MI, in the behavioral accuracy of MI, and in electroencephalographic variables, were able to differentiate between high- versus low-aptitude BMI users. High-aptitude BMI users showed higher MI accuracy as captured by subjective and behavioral measurements, pointing to a prominent role of kinesthetic rather than visual imagery. Additionally, for the first time, we applied mental chronometry, a measure quantifying the degree to which imagined and executed movements share a similar temporal profile. We also identified enhanced lateralized μ-band oscillations over sensorimotor cortices during MI in high- versus low-aptitude BMI users. These findings reveal that subjective, behavioral, and EEG measurements of MI are intimately linked to BMI control. We propose that poor BMI control cannot be ascribed only to intrinsic limitations of EEG recordings and that specific questionnaires and mental chronometry can be used as predictors of BMI performance (without the need to record EEG activity).

Non-invasive brain machine interfaces (BMI) have enabled humans to control a variety of external devices through real-time decoding of brain activity. In non-invasive BMI approaches, users are engaged in a cognitive task, such as visual attention or motor imagery (MI), while their brain signals are recorded through electroencephalography (EEG) and on-line processed to ultimately control external devices^1^,^2^. Despite important developments concerning both recording and analysis techniques of brain signals, the ability to control MI-based BMI is characterized by large inter-individual differences. The portion of users that cannot achieve successful control is usually reported to be in the range of 15–30%^3^. However, studies employing large number of subjects have shown that up to 50% of users are not able to achieve accuracy above 70%, a threshold commonly considered more accurate for identifying successful control^4^,^5^.

Previous work using MRI and EEG recordings have identified anatomical (volumetric and connectivity measurements)^6^,^7^, functional (extent of the cortical activation)^8^, as well as electrophysiological indices (power modulation over specific frequency bands)^9^,^10^,^11^ as potential factors for this variability in BMI-control. In addition, psychological, cognitive and motor characteristics have been evaluated. These include (i) questionnaires aimed at the self-assessment of personality and psycho-pathological traits^12^,^13^, (ii) individual intrinsic characteristics such as age and the per-day amount of hand-arm movement^14^, and (iii) behavioral tasks assessing motor and cognitive skills, such as visuo-motor coordination and concentration^15^.

However, the specific ability to perform the particular cognitive task required for MI-based BMI, i.e. motor imagery (MI), has not been investigated in detail. Although different self-reporting motor imagery questionnaires have been employed in the context of BMI^7^,^12^,^16^,^17^, these have led to inconsistent results. It should be noted that as with BMI control skills, MI ability, that is the ability to mentally perform or rehearse an action without actually executing it (see for reviews)^18^,^19^, also presents marked differences across the general population. These differences have been extensively evaluated in the field of cognitive neuroscience using subjective methods such are the aforementioned self-reporting motor-imagery questionnaires, but also through different behavioral measures. These include objective measurements assessing the relationship between the duration of executed and imaged movements, such as the mental chronometry task^20^,^21^,^22^. In addition, the effects of MI translate into physiological (e.g. skin conductance, heart beat variation and respiratory rate) and neurophysiological modulations that can be evaluated with neuroimaging techniques^23^ and transcranial magnetic stimulation^24^.

In the present study we directly addressed whether the variability in the ability to control a BMI and in performing MI are linked in the sense that in MI-based BMI paradigms, variations in BMI control performance can be related to the different individual skills in MI. Better understanding this potential relationship might elucidate whether BMI and MI rely on common mechanisms and whether MI ability as quantified by measures from cognitive neuroscience may be a predictor of BMI-performance. We investigated MI accuracy in high- and low-aptitude BMI users and used a reliable behavioral task that has previously been employed to collect an objective (mental chronometry) measure of MI ability^21^,^25^. We additionally used a self-reporting questionnaire with two sub-scales that separately assess kinesthetic and visual features of MI^26^. If variations in BMI control performance are associated with the different individual skills in MI, we predicted to find better motor MI performance (mental chronometry task) and higher MI scores in the questionnaires in high-aptitude rather than low-aptitude BMI users. Kinesthetic MI has previously been shown to activate motor-associated brain structures^23^ involved in BMI control^8^ to a greater extent than visual MI. Therefore, we tested the hypothesis that kinesthetic MI would be more strongly associated with BMI aptitude as compared to visual MI. Finally, we compared neural MI correlates extracted from EEG signals in high- and low-aptitude BMI users. Since BMI-based control has been related to specific lateralized activity in bilateral motor and premotor cortices^8^,^27^, we hypothesized that the lateralization of cortical activity recorded during pure MI (that is without visual feedback as during BMI control) would discriminate between high- and low-aptitude BMI users.

## Results

In order to study the relationship between BMI proficiency and MI, we first classified participants (N = 24) as high- vs. low-aptitude BMI users, depending on their performance during the BMI task. In the MI task, participants were asked to move a cursor to the left or right side of a computer screen by imagining, respectively, left or right hand clasping movements. Based on previous BMI studies^6^,^28^, we divided our pool of subjects into two groups according to the median value (69.5%) of the overall on-line BMI control performance. For each subject, the mean BMI performance was computed as the percentage of time frames in which the cursor moved in the cued direction. The low aptitude group consisted of 12 subjects, with performance ranging from 49% to 67%, while the high-aptitude group had a performance ranging from 72% to 87%. We note that the threshold around 70% of accuracy is in accordance with that previously proposed by others authors to discriminate effective BMI-control from chance in a 2-class paradigm^5^,^28^.

### MI behavioral abilities and questionnaire discriminate between high- and low-aptitude BMI users

MI ability was evaluated through two different measurements: the mental chronometry task^29^, assessing the difference in duration between actual and imagined movements and a self-reporting questionnaire, the MIQ-RS^26^, including two subscales measuring visual and kinesthetic features of MI. The mental chronometry task is considered a reliable measure of MI quality and aims at assessing individual’s ability to preserve the temporal characteristics of the movement during MI, based on the isochrony between real execution and MI^24^,^29^.

#### Questionnaire

**Figure 1:**
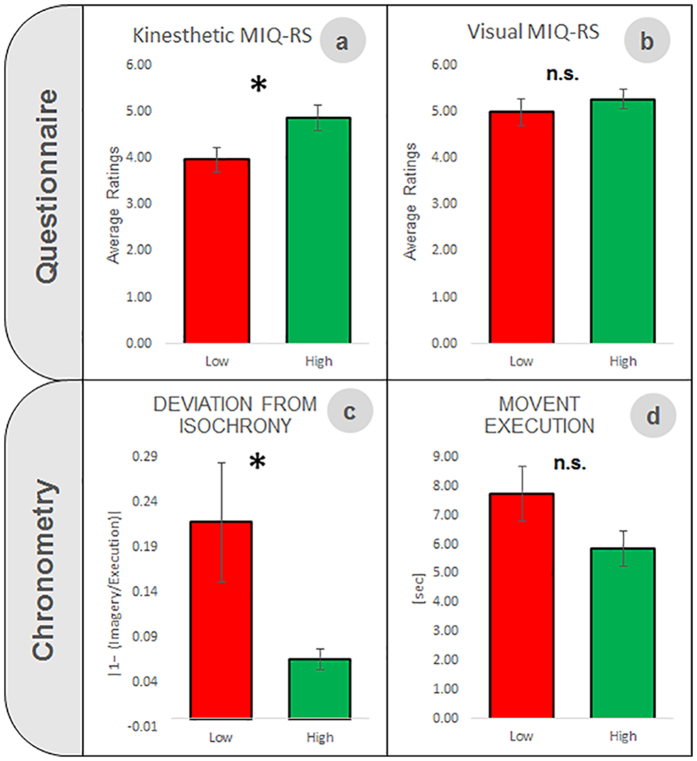
Significant differences between high- and low-aptitude users in motor imagery abilities evaluated by using a MI questionnaire and mental chronometry. Average ratings (±standard error) among all items of the *MIQ-RS questionnaire* separately for the visual and kinesthetic subscales (upper panels). High aptitude users rated *kinesthetic* motor imagery as significantly easier than low aptitude users **(a)**, while no difference between the groups was found for the visual MI subscale **(b)**. Results (mean values ± standard error) related to the chronometric measurements are shown in the bottom panels. The *Deviation from isochrony* index (i.e. the deviation from an equal time required to execute and to imagine five hand clasping movements) was worse (higher) in low rather than high aptitude users **(c)**. Conversely, the average time required to execute the movement did not differ between the two groups (**d**). Data in (**c**,**d**) are collapsed across left and right hands since they did not statically differ.

The MIQ-RS questionnaire showed that for high aptitude users, the kinesthetic experience of MI was judged as more vivid and easier to evaluate than for low aptitude users, as shown by higher ratings in the former group in the kinesthetic MI scale (t = −2.255, p = 0.034, Fig. 1(a)). No difference between the groups was found for visual MI scale (t = −0.771, p = 0.449, Fig. 1(b)).

#### Mental chronometry

Based on the MI task used for the BMI control, we compared the time required to execute and imagine five-hand clasping movements. The temporal congruency between MI and execution is an indicator of good imagery abilities, whereas a higher discrepancy between the two modalities have been associated with poor imagery quality. The repeated measure ANOVA with *imagined hand* (left versus right), *task* (execution versus MI) and *group* (high- versus low- aptitude) as factors, performed on the mental chronometry data revealed a significant difference in the duration of the MI task, but, importantly, not in the execution task, in the two groups (*task* X *group*, F(1, 22) = 4.383, p = 0.048). Post hoc comparisons (Newman-Keuls tests) showed that in low-, but not in high-aptitude BMI users the imagery of hand clasping movements takes longer than the execution of the same movement regardless of the employed hand (low-aptitude BMI users p = 0.002; high-aptitude BMI users: p = 0.619). In contrast, the result in high-aptitude users is compatible with the notion of *isochrony* (see next paragraph). Interestingly, the duration of the execution was equal in the two groups and for both hands (p > 0.05, Fig. 1(d)).

In addition, we calculated an index of *deviation from isochrony*, in order to quantify the discrepancy from the temporal congruence (*isochrony*) in the duration of imagined and executed movement (for further details on the calculation, refer to *Materials and methods)*. Low values of this index indicate high MI quality, in accordance with the fact that good imagery skills are associated to reduced differences between the time required to execute and imagine the movement^21^,^24^,^29^. The t-test performed on the *deviation from isochrony index* revealed a significant difference between groups (t = 2.175, p = 0.041, Fig. 1(c)), with values close to zero in high-aptitude users and greater values in low-aptitude ones, indicating a link between higher MI accuracy and better BMI control abilities.

### EEG data of MI discriminate between high- and low-aptitude BMI users

**Figure 2:**
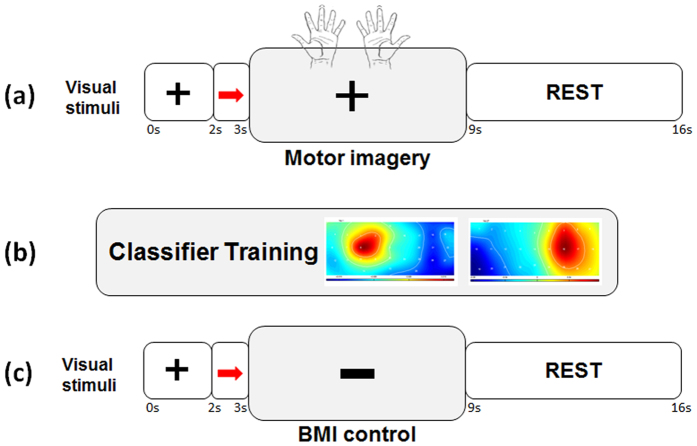
Experimental procedure during the BMI session. **(a)** *Calibration session, off-line*: subjects were instructed to imagine clasping movements performed either with the left or the right hand as indicated by a visual cue (red arrow) without receiving any visual feedback. **(b)** The collected EEG data were then used to extract EEG correlates of motor imagery, to train a linear classifier and to extract subject specific spatial filters. **(c)** These parameters were then used in the *on-line session*, during which subjects were asked to control in real-time the movement of a cursor (black rectangle) by performing the same motor imagery task as in the calibration session.

In order to compare electrophysiological correlates of MI abilities in low- and high-aptitude BMI users, we analyzed EEG signals collected during pure MI without BMI (with no visual feedback, nor cursor control), at the beginning of each session (*calibration session*, off-line, see Fig. 2(a)). We used two analyses: we analyzed the modulation of the μ-rhythm and we exploited the information contained in the common spatial patterns (CSPs).

#### μ-rhythm

**Figure 3:**
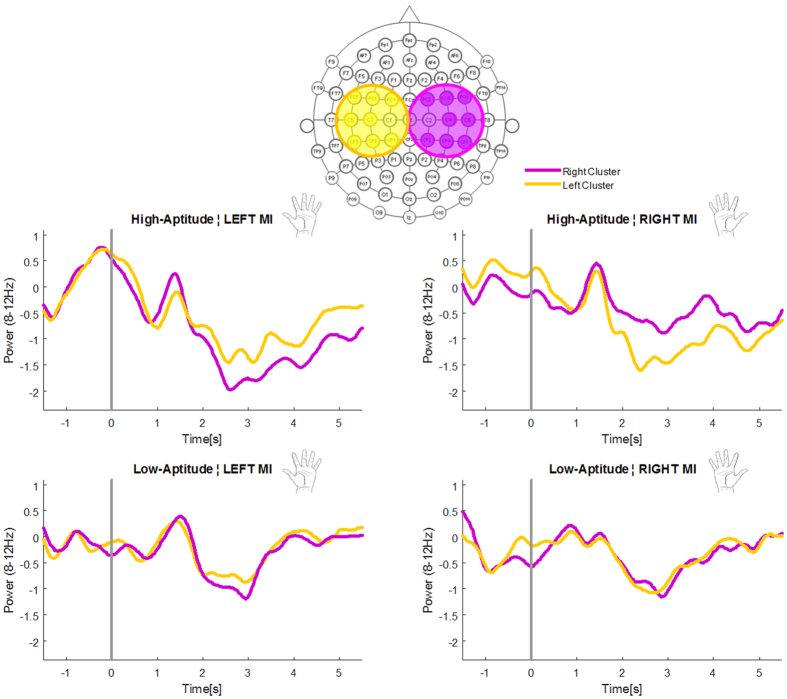
μ-rhythm suppression during motor imagery over two sensorimotor clusters. The panel illustrates the mean power spectrum (±standard error across subjects) in the μ-band (8–12 Hz) over two clusters of electrodes located over sensorimotor regions in the left (yellow) and the right (purple) hemisphere during left (on the left) and right (on the right) hand motor imagery. Traces in the contralateral cluster with respect to the imagined hand show a clear μ-rhythm suppression, stronger in the high-aptitude (upper plots) as compared to the low-aptitude (lower plots) group. Conversely, the mean power over the ipsilateral cluster presents a weaker suppression with respect to the contralateral cluster. These effects have been quantified with the proposed *index of laterality*.

**Figure 4:**
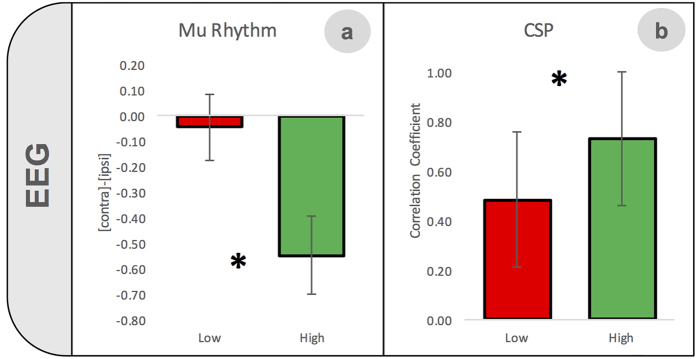
Significant differences between high- and low-aptitude BMI users in EEG-correlates of pure motor imagery. The difference in μ-rhythm suppression (mean values ± standard error) between two clusters of electrodes, one over each hemisphere (*index of laterality*) was significantly more marked in the high- than in the low-aptitude group, showing the importance of a specific lateralized activation during MI for BMI control (**a**). The correlation (mean values ± standard error) between the right and left common spatial patterns (CSP) was higher in high- rather than low-aptitude users, indicating a more lateralized cortical activity during MI in the former group (**b**).

In order to investigate differences in μ-rhythm power spectra between high- and low-aptitude BMI users in all subjects we considered a fixed frequency range (8–12 Hz). This range over sensorimotor regions has been classically referred to as the μ-rhythm, and it has been consistently reported to be modulated during motor imagery (see for instance^30^). We selected two clusters of electrodes located over sensorimotor regions in each hemisphere (Fig. 3). We considered the average value of each cluster, separately for the two imagined hands. Finally, an *index* of *laterality* was computed considering the difference between the contralateral and ipsilateral cluster, for both hands. A repeated measure ANOVA with factors *imagined hand* (right and left) and g*roup (*high and low-aptitude BMI users) run on the *index* of *laterality* revealed a main effect of group (F(1, 22) = 5.778, p = 0.025), independently of the imagined hand (imagined hand, F(1, 22) = 0.326, p = 0.574; imagined hand X group, F(1, 22) = 0.213, p = 0.649, Fig. 4(a)). Thus, irrespectively of the hand involved in MI, high-aptitude users showed a more negative index of laterality compared to low-aptitude users (group, F(1, 22) = 5.778, p = 0.025). Note that a more negative value corresponds to a stronger difference between the contralateral and the ipsilateral activity, in the direction of a more marked suppression over the contralateral hemisphere. Thus, these results reflect a higher difference in μ-rhythm suppression in the hemisphere contralateral to the imagined hand (with respect to the ipsilateral one) in high- as compared to low-aptitude BMI users, as depicted in Fig. 3. We note that our choice of the electrodes selected to be included in the two clusters was further corroborated by results at the level of single channels (uncorrected). High-aptitude users showed more strongly suppressed activity over electrodes located in the hemisphere contralateral to the imagined hand. More specifically, for right-hand MI, we found a statically significant effect over electrodes FC5, FC3, C5, C3, CP5 and CP3, while for left-hand MI, electrodes FC2, FC4, FC6 and C4.

#### CSP analysis

CSPs are subject specific spatial filters that weigh each electrode according to its contribution to the classification^31^. We expected to identify a stronger contribution from those channels which are located in the hemisphere contralateral to the imagine hand (Supplementary Figure 1). To evaluate the presence of this lateralized pattern, we computed the correlation coefficient between one CSP referring to right-hand MI (first CSP) and the mirrored-image of one CSP relative to left-hand MI (last CSP). Thus, if this lateralized pattern is associated to a more proficient modulation of neural activity for both hands during MI, the correlation coefficient should be higher in high-aptitude BMI users. The CSP analysis showed that in high-aptitude BMI users, the first and last CSPs were more strongly correlated (t = −2.153, p = 0.043) than in low-aptitude BMI users (Fig. 4(b)), suggesting a higher lateralization during MI in the high-aptitude group.

### Prediction of BMI-control abilities

Given the significant difference between high- and low-aptitude users on the kinesthetic sub-scale and the mental chronometry task, we next used a linear classifier (logistic regression algorithm) to investigate whether these subjective and behavioral indices are potential predictors of BMI-control ability. More specifically, we tested how accurately the estimated model would predict the assignment of each individual subject to either the high- or low-aptitude BMI group. This analysis lead to an average accuracy of 73.7% of correct classification and was above the threshold derived with a binomial cumulative distribution for a two-class classification problem^5^,^32^ (70%; sample size: 24 subjects). The control analysis, performed using as predictors the ratings on the visual subscale of the MIQ-RS and the duration of executed clasping movements acquired during the mental chronometry task, resulted in a lower average accuracy (57.1%), that it is below the significant threshold obtained with the binomial cumulative distribution.

## Discussion

Our study aimed at investigating whether MI ability underlies proficiency in BMI control. We found that EEG signals during MI (as captured by μ-rhythm and CSP) as well as behavioral and subjective measurements of MI accuracy (mental chronometry; kinesthetic sub-scale of the MIQ-RS questionnaire) distinguished between users with high- and low-aptitude for BMI control. In particular, high-aptitude BMI users showed higher MI accuracy and more lateralized neural activation during MI as compared to low-aptitude BMI users. In addition, our results suggest that questionnaire and behavioral measures can be used as predictors of BMI performance, without the need for recording EEG signals.

### Higher motor imagery ability in high-aptitude BMI users

We used two reliable cognitive indices of MI abilities: self-reporting scores concerning kinesthetic features of MI activity (based on MIQ-RS questionnaire) and an objective behavioral measure based on mental chronometry. Both measures exhibited a significant difference between the high- and the low-aptitude group. We will discuss the subjective questionnaire data first. In the context of our chosen questionnaire, higher ratings indicate an ease in performing the task. Kinesthetic MI ratings from proficient BMI performers were significantly higher than those from the low-aptitude group, whereas there was no such group difference for subjective ratings of visual MI, highlighting the importance of motor and kinesthetic imagery (participant’s ability to *feel* imagined movements) rather than visual imagery (participant’s ability to *see* imagined movements) in BMI control.

Different MI questionnaires (e.g. Vividness of movement imagery questionnaire in^12^,^17^; the revised Movement Imagery Questionnaire in^7^; The Kinesthetic and Visual Imagery questionnaire in^16^) have already been employed in the context of evaluating BMI abilities. However, to date only one study reported a significant relationship between BMI performance and imagery scores for visual versus kinesthetic modalities^16^. In line with our results, Vuckovic and collaborators^16^ found a stronger correlation between participants’ ratings in kinesthetic versus visual MI. Crucially, they considered the accuracy of an off-line EEG classifier using data recorded during MI, but not during BMI control as in the present study. Our study significantly extends that finding in showing the prominent role of kinesthetic imagery in explaining inter-subject differences, not only during MI, but also during on-line BMI control. We note that previous studies had underlined the importance of instructing subjects to perform kinesthetic rather than visual motor imagery in BMI paradigms^9^,^34^. Moreover, kinesthetic MI activates similar sensorimotor areas, whose activity is classically decoded for MI-based BMI, namely primary motor^35^,^36^, SMA, and ventral premotor cortex^23^. Compatible with the present discrimination between high- and low-aptitude BMI users in kinesthetic versus visual MI, visual MI fails in producing a clear activation in these areas^37^ and activates more posterior regions in occipital and parietal cortex^23^.

Importantly, the present study is the first to use mental chronometry to investigate difference in BMI ability. We note that MI questionnaire, despite being a validated tool in MI and previously used in BMI ability estimation, reflect a subjective assessment of MI abilities based on verbal rating scales. In contrast, mental chronometry is an objective behavioral measurement based on motor responses and thus less prone to participant biases: for this reason it has been argued to be a more reliable criterion of MI quality, compared to questionnaire scores^21^. Results from the mental chronometry task revealed that low-aptitude BMI users (as compared to high-aptitude users) show a greater difference between the time required to perform versus imagine a hand clasping movement, indicating reduced MI abilities. This interpretation is supported by an extensive body of literature showing that poor *isochrony* is an indicator of impaired imagery abilities, both in healthy participants^21^ and in different patient groups^19^,^38^,^39^,^40^. These behavioral results demonstrate that the *isochrony* in MI is also relevant to BMI proficiency, suggesting that higher MI abilities are crucial for BMI control. Thus, we introduce here the mental chronometry and the kinesthetic questionnaire sub-scale as predictors of BMI control; this is particularly interesting as especially the latter measure targets specifically MI skills in a reliable way without the necessity for EEG recordings. For instance, by employing the chronometry index it is possible to evaluate and monitor MI abilities in different populations (healthy adults; patients), and over various experimental conditions or phases of training or illness. Results from the mental chronometry analysis also highlighted that the duration of the executed movement per se seems not to influence BMI ability. These data further extended prior cognitive neuroscience works by showing that not only MI but also BMI control does not exclusively depend on the ability to actually perform the movement, but also relies on the complex interplay between motor abilities and somatosensory inputs from the body part involved in the MI task^41^,^42^.

Despite having instructed our participants not to perform any overt movement during motor imagery and despite close observation by the experimenter, we cannot exclude that sub-threshold movements not detected by us could be present or that spinal cord circuitry could be still activated. Concerning, spinal circuitry involvement, previous studies in primates indeed have shown activity at the spinal level during movement preparation (e.g.^43^). Moreover, in humans, a residual muscular activity and spinal cord modulation have been reported during motor imagery in some subjects and under certain conditions, as a variation of inhibition at level of spinal interneurons^18^. Although not likely, such potentially undetected movements and spinal cord activity could have differentially impacted BMI performance in the high- and low-aptitude group during motor imagery. However, it is improbable that these effects, if present, could account also for the differences found in our subjective and objective measurements of MI ability between the two groups. Previous studies, indeed, already reported dissimilar scores in good and poor imagers at the mental chronometry task and at the MIQ-R, that were not explained by residual muscular activity (e.g.^24^).

### EEG-signatures of motor imagery differ in high- and low-aptitude BMI users

To investigate neural signatures that might reflect differences in hemispheric lateralization during MI between low- and high-aptitude users, we found two further indices derived from EEG signals during MI without online BMI control. First, we compared the difference in μ-band suppression in two clusters of electrodes over the sensorimotor cortices and found that the laterality index was larger in the high-aptitude users compared to the low-aptitude group, pointing to the importance of the lateralization of a specific frequency band during MI for BMI control. This conclusion was further supported by another analysis, aimed at evaluating the neurophysiological information carried by the CSP (*CSP index* computed as the correlation coefficient between the right and left CSP, separately for each subject). Results showed that in high-aptitude BMI users, the *CSP index* was significantly higher than in low-aptitude users, indicating that electrodes that discriminate the most between imagery of left and right hand movements are located over the contralateral hemisphere. This confirms that this lateralized pattern of neural activity, weaker in low-aptitude BMI users, is important to achieve good BMI-control.

Previous BMI studies have extracted predictors able to differentiate between high- and low- aptitude BMI users based on EEG signals recorded not only during BMI-control, but also at rest. For instance, an index of the average alpha power recorded at rest over both C3 and C4 has been shown to be related to BMI performance^9^. Other studies focused on the importance of different frequency bands, involving sensorimotor gamma oscillations during MI^44^, or negative correlations between BMI performance and theta band activity at rest^10^. Similarly even during on-line BMI control, a recent study showed that, the association between a laterality index extracted from two central EEG electrodes (computed as a difference between the μ-rhythm desynchronization over C3 and C4) and the BOLD signal acquired during simultaneous fMRI recordings, could be used to group BMI users into good, moderate and poor performers^27^. Other studies focused on the importance of different frequency bands, involving sensorimotor gamma oscillations during MI^44^, or negative correlations between BMI performance and theta band activity at rest^10^. Structural brain features such as interhemispheric connections across the corpus callosum^45^ have also been proposed to be important for MI-based BMI^6^. The present data corroborate the differential involvement of the two hemispheres previously reported during BMI control or at rest in high-aptitude users. Our data suggest that this pattern in good BMI users is related to MI, showing that different lateralization during MI discriminates between high and low BMI proficiency.

These findings suggest that the ability to perform the underlying cognitive task (MI) important for BMI control, is mediated via bilateral connections driving proficient BMI aptitude. Literature on MI again offers support for this proposal. During MI, a power decrease over the contralateral hemisphere (event-related desynchronization, ERD) is often associated with a power increase (event-related synchronization, ERS) over the ipsilateral hemisphere^46^. Other studies^47^,^48^ investigated the reciprocal influence of both hemispheres during MI, and the inhibition of the contra- versus ipsilateral hemisphere has been described in good MI subjects^49^. Moreover, this effect during MI mimics the pattern of lateralization largely documented for actual unilateral movements: during the execution of unimanual movements, the contralateral motor cortex inhibits the ipsilateral one through the transcallosal pathway, inducing a decrease of ipsilateral M1 excitability^49^. Similarly, directionally selective disruptions of oscillations have been also documented during movement preparation in primates^51^,^52^. Here we extend these neuroscience findings to the field of BMI by showing that MI-based BMI control depends not only on the modulation of the contralateral hemisphere, but more importantly, on the differences between the activation in the two hemispheres in a specific frequency band (i.e. the μ-rhythm).

Finally, results from the logistic regression analysis show the potential of our behavioral measures, not only in explaining differences between high- and low-aptitude BMI users, but also in predicting BMI control ability at the individual level. The scores on the kinesthetic scale of the MIQ-RS questionnaire and the index of *isochrony* on the mental chronometry task were indeed significantly above chance in classifying individual participants as high- vs. low-aptitude BMI users, whereas this was not the case for control analysis (including execution of movement measurements and visual imagery sub-scale scores). A model based on these two MI indices might present a powerful tool for screening and monitoring BMI abilities; moreover these measures do not require collecting and analyzing EEG data to estimate BMI aptitude. We note that a previous study directly investigated the use of psychological variables as predictors of BMI proficiency^12^. At present, we cannot exclude that the skills underlying the tests used by these authors might be involved in the ability to perform MI or somehow relate to the tasks we employed here (some of the tests used may relate to motor prediction^53^ and concentration^54^ that are both linked to MI). Further research will be required to directly test these issues.

## Conclusions

Taken together our results suggest that subjective, behavioral, and EEG measurements of MI ability are intimately linked to the ability to control a BMI. The finding that MI ability predicts BMI aptitude provides evidence against the hypothesis that BMI illiteracy can be completely ascribed to intrinsic limitations of EEG in recording brain signals, in particular due to specific neuroanatomical characteristics^27^. We cannot exclude that these technical limitations might have an impact on BMI proficiency, however the present results reveal the importance of a specific cognitive ability - MI - in BMI control. The proposed pre-screening tool based on subjective and behavioral MI measurements aims not at excluding low-aptitude BMI users, but rather at adopting specific strategies to improve their MI abilities, and thus potentially their BMI proficiency. Different aspects can influence the ability to perform MI, among them being posture^55^,^56^, perspective^57^,^58^ and the type of MI (visual or kinaesthetic)^37^,^58^. Past MI research has proposed different strategies for improving motor imagery skills in athletes^59^ and even in stroke patients^38^, by providing, for instance, verbal information describing the sensation elicited by a given movement^59^, by presenting objects that can stimulate specific affordances and sensations or by using action observation prior to the imagination task^38^. The present data show that these neuroscience-based strategies can now also be applied to BMI- “athletes”, aiming at activating the motor representation related to the action to be imagined, thus facilitating MI, which, based on the present results, might improve BMI control.

## Materials and Methods

### Participants

Twenty-four right-handed (as determined by the Handedness Inventory)^61^ healthy participants (7 females and 17 males, mean age 26.5 years, SD ± 3.8, range 21–34) took part in the experiment which was approved by the Ethics Committee of the Faculty of Biology and Medicine of the University of Lausanne and was conducted in accordance with the Declaration of Helsinki. Written informed consent was obtained from all participants that took part in the experiment.

### Experimental procedure

Two different experimental sessions were run. One session was dedicated to the EEG recording and BMI-control while the other aimed to behaviorally assess MI ability. The order of the two sessions was counterbalanced across subjects.

#### Motor imagery ability

##### Questionnaire

The MIQ-RS^26^ is composed of two subscales aiming at evaluating participant’s ability to see (visual imagery) and feel (kinesthetic imagery) different imagined movements.

Every subscale contains seven items corresponding to seven different movements (e.g. pushing, pulling, reaching and grasping object, make a fist).

Each item entails first to perform a movement, then to imagine the same action, and finally rate the difficulty of the imagination task using a 7-point scale (from 1 = very hard to see ⁄ feel; 4 = neutral (not easy⁄not hard); 7 = very easy to see⁄feel, and intermediate levels). Questionnaires have been commonly used to asses motor imaginary ability^21^. The MIQ-RS is a suitable option for examining movement imagery abilities targeting primarily the upper extremities^26^.

##### Mental chronometry

Here in accordance with the BMI task, subjects were asked to perform or imagine five hand clasping movements, either with the left or the right hand. They were instructed to imagine the feeling of performing the movement (kinesthetic MI) with eyes open as in the BMI task. There were in total 4 experimental conditions, with hand (left/right) and task (execution/imagination) as factors. Each condition was repeated 10 times to account for a reliable estimation of the actual and imaged movement duration.

We used a chronometer to measure the time required to complete the 5-hand clasping task. Subjects were instructed to verbally report the moment in which they start and complete the task.

The rationale of the mental chronometry task is based on the evidence that imagined and executed movements follow the same rules of motor control^22^,^39^ and that a temporal congruence exists between the duration of executed and imaged action. For this reason, comparing duration of motor imagery and of the real execution of the same action has been previously employed as a simple and reliable method to evaluate imagery accuracy in healthy participants and in patients^21^,^24^,^38^,^41^.

#### EEG acquisition and BMI loop

A 64-channel EEG system (g.tec medical engineering GmbH, Graz, Austria) was used for the EEG recording. A subset of 27 of a total of 64 available EEG channels were considered for the signal recording and the BMI loop, which refers to the processing chain from the decoding of brain activity to the feedback presentation. This subset included all the electrodes situated over the sensorimotor cortex, including fronto-parietal, central and centro-parietal electrodes. The ground was located on the forehead (AFz) and the reference was placed on the right earlobe. The EEG signal was sampled at 256 Hz and Butterworth filters (in the range of 1–30 Hz) were used during the recording.

**Figure 5:**
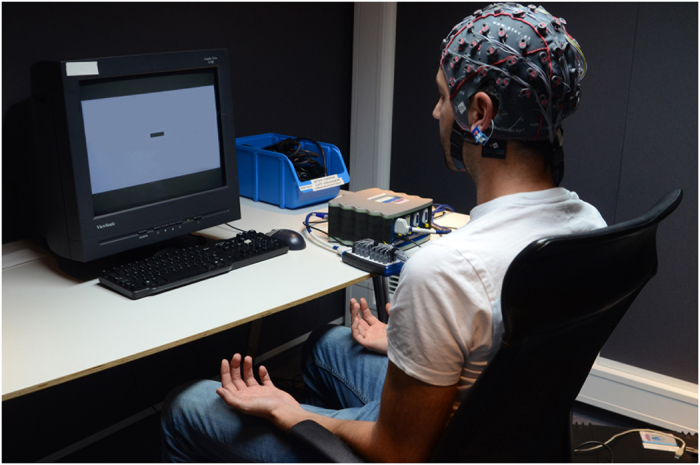
Experimental setup during EEG acquisition and BMI-control: A 64-channel EEG system was used for recording brain activity. During motor imagery and BMI control, the instructions and the visual stimuli, including the BMI feedback as shown in the picture, were presented on a computer screen. Participants sat comfortably in front of the screen, keeping their hands on their thighs with the palms up.

During the EEG-BMI session participants sat comfortably in front of a computer screen onto which the stimuli and BMI visual feedback was projected. They were instructed to keep their hands on their thighs with the palm facing up (Fig. 5) and to avoid any movement during the motor imagery and BMI-control. The whole experiment included two off-line (*calibration session*, see below) and two online (*BMI cursor control*, see below) runs.

#### Calibration session: pure motor imagery and classifier training

Each recording began with the two off-line runs of 40 trials each, during which the subject performed MI without visual feedback while the EEG signal was recorded. Subjects were instructed to imagine clasping movements performed either with the left or the right hand by focusing on the kinesthetic sensations of the movement (kinesthetic MI). The trial began with a fixation cross (2 seconds), followed by a red arrow (cue, 1 second), placed in the center of the screen, indicating which hand had to be imagined. Right after the cue disappeared, the subject was instructed to start imagining the movement and continue for a total duration of 6 seconds (Fig. 2(a)). After the motor imagery period, subjects had 7 seconds of rest.

The off-line runs were required in order to select subject-specific discriminant EEG features between the left and right hand imaged movements, namely the spatial filters and the linear classifier parameters to be used during the on-line control session.

The spatial filters (common spatial patterns, CSPs), were used to improve the efficiency in the discrimination of two classes (left and right hand imaged movement) and represents a method commonly used in BMI applications to increase the signal strength^31^. The CSP algorithm finds, for each subject, spatial filters (in the same number as the electrodes), each of which weights each electrode based on its contribution to the classification (Supplementary Figure 1).

In order to compute the classifier and extract the CSP, the EEG data acquired during the offline session were first visually inspected to exclude trials that might contain artifacts. Then the EEG signal were band-pass filtered between 8 and 30 Hz, to include the μ (8–12 Hz) and β (12–30 Hz) frequency bands. After this, the signal’s matrix of covariance was computed: the CSP algorithm works by maximizing the variance for one class and at the same time minimize variance for the other class. The variance of the band-pass filtered signal is equivalent to the band power of the signal and the signal variance in the frequency band exploited for the BMI directly reflects the presence of the typical motor-imagery-related patterns of activity. Based on previous studies^31^, we expected the firsts two CSPs to show a prominent contribution from electrodes located over the hemisphere contralateral to one imagined hand movement, and likewise for the last two CSPs, but for the other hand.

For each subject, the first and last two CSPs are used to construct four feature vectors^31^ and these feature vectors are then used to build the linear classifier (LDA classifier, Fig. 2(b)).

#### On-line session: BMI cursor control

During the on-line session subjects were instructed to mentally perform left or right hand clasping movements as in the calibration phase but with the aim of attempting to control the movement of a cursor (black rectangle) towards the cued side of the screen.

At each time frame, the output of the linear classifier provided the direction and the velocity for the cursor displacement.

As in the *calibration session*, each trial began with the presentation of a fixation cross (2 seconds) followed by a central arrow, (1 second, cue), pointing either to the left or to the right, indicating which hand had to be imagined (right or left) and the side of the screen towards which the subject should attempt to move the cursor (Fig. 2(c)). The hand involved in the imagined movement and the cued side of the screen were always coincident (e.g. right hand and right side of the screen). After 1 second, the cursor appeared and the subject could start immediately controlling it.

If the cursor reached the side of the screen within the timeout period of 6 seconds, then the cursor disappeared and a fixation cross was displayed for the remaining time. At the end of the cursor control period, subjects had a 7 seconds of rest. The total duration of each trial was 16 seconds. There were a total of 64 trials divided into two runs, equally divided into the two imagined hand.

### Data Analysis

#### Analysis of behavioral data

##### Questionnaire

For each subject, we computed the *average of the ratings for visual and kinesthetic MI items* of the MIQ-RS, separately. Then, we statistically tested for differences between the two groups (independent samples t-test, two-tailed), independently for the two subscales.

##### Mental chronometry

Therefore, we computed for each subject the *average time* required to perform the actual or imaged clasping movement with the right and left hand in the mental chronometry task. We submitted these values to a repeated measure ANOVA with *imagined hand* (left versus right) and *task* (execution versus MI) as within-subjects factors and with *group* (high versus low-aptitude BMI users) as between-subjects factor.

Additionally, we calculated an index of *deviation from isochrony* by computing the ratio between the average duration of actual and imaged clasping movement for every subject and finally by considering the absolute value of the deviation of this ratio from 1, as indicated by the following formula:


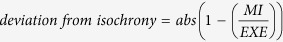


A ratio equal to 1 (“*isochrony*” between MI and actual execution) indicates an equal time required for completing both tasks. In agreement with the “principle of isochrony” governing the relationship between imagined and actual movement duration^21^,^22^,^39^, higher values of “deviation from isochrony” correspond to lower MI abilities. We then compare statistically this index between the two groups (independent samples t-test, two-tailed).

### Statistical analysis of electrophysiological data

#### Analysis of μ-rhythm

First we computed for each subject and separate for the right and left MI, the event shift in the μ band (8–12 Hz) power spectrum (event related spectral perturbation, ERSP^61^), considering as a baseline the first two seconds at the beginning of each trial, during the presentation of the fixation cross and the arrow indicating which hand to imagine. Then we considered the average over time from 1 seconds after the beginning of the MI period till the end of the 6 seconds of MI.

We first investigated the presence of the classical modulation of the μ-rhythm due to MI already reported in an extended body of literature^46^. Then, as an exploratory approach we statistically tested for differences in the μ-band desynchronization between the high- and low-aptitude groups, separate for left and right MI over all electrodes, except for those lying along the central line and those over fronto-temporal, temporal and temporo-parietal regions. We then selected two subgroups of electrode, one over each hemisphere, and extracted the average value over each cluster for the two imagined hands (Fig. 3).

We computed an *index of laterality*, representing the different degree of activation between the two hemispheres: to do this we calculated the difference between the contralateral and ipsilateral cluster separate for the two imagined hands. This gave us a single index accounting for the difference in activation between the hemisphere contralateral to the imagined hand and the ipsilateral one over sensorimotor regions. We expected a more pronounced difference, i.e. a higher lateralization between the two hemispheres, in high aptitude users. More specifically, since the μ-band modulation is expressed in term of desynchronization with respect to the rest period at the beginning of each trial, we expect a stronger suppression, thus a more negative value, for high aptitude users than for low-aptitude users. We run a repeated measure ANOVA with *imagined hand* (right versus left) as within-subjects factors and with *group* (high versus low-aptitude BMI users) as between-subjects factor. If the ANOVA revealed a significant interaction, we performed post hoc comparisons using Newman-Keuls tests.

#### Analysis of Common Spatial Pattern

We decided to investigate whether the different performance between the two groups could be depicted by the topography of the first and the last CSP. This analysis was motivated by the observation that a high aptitude BMI user would likely present a clear lateralized pattern of activation (Fig. 2(b) and Supplementary Figure 1), with a more prominent contribution in the classification from electrodes placed contralateral to the imagined direction as already described elsewhere^31^. We computed then the correlation coefficient between the first CSP (right hand MI) and the mirrored-imaged of the last CSP (left hand MI): if the lateralized pattern would be present in both CSP, it would result in a higher correlation coefficient. Then, we statistically investigated (independent samples t-test, two-tailed) differences in the correlation coefficient between high and low-aptitude BMI users.

### Prediction of BMI-control abilities

Moreover, we were interested in investigating whether the two behavioral indices related to MI abilities could also be exploited to predict if a subject would belong to the high- or low-aptitude group. Behavioral, rather than the neurophysiological data are investigated as predictors for two reasons. First, we wanted to avoid any possible circularity which could be caused by using as predictors, variables extracted from EEG data, being the division between high and low-aptitude users based on the BMI-control performance.

Secondly, behavioral predictors offer the great advantage of avoiding setting up an EEG recording, thus notably decreasing the acquisition time.

Thus, we used two behavioral variables extrapolated from the kinesthetic ratings to MIQ-RS questionnaire and from mental chronometry task (deviation from isochrony) as predictors for a linear classifier based on a logistic regression algorithm^62^. The training and testing of the classifier were performed using a 8-out cross validation procedure, in which the training set was chosen by leaving out, for the test set, the data from 4 subjects per group. Thus, since our pool included 24 participants, the training set was composed by 16 subjects. This division and the corresponding classification was iterated several times to account for all the possible combinations of training and test set (exhaustive cross-validation). The empirical accuracy was computed by averaging the single accuracy from every cross validation loop.

We tested the statistical significance of our accuracy by considering the binomial cumulative distribution. The binomial distribution give us an analytical threshold of the statistical significance for a two-class classification problem: only in case of an infinite sample size, the statistical significant threshold corresponds to the theoretical chance level at 50%, otherwise the real chance level depends on the sample size (see for a discussion about this topic^32^).

To provide a further evidence for the relevance of our behavioral variable in predicting BMI proficiency, we performed the same analysis using two control variables: ratings of the visual subscale of the MIQ-RS and the duration of executed clasping movements acquired during the mental chronometry task (computed as the average duration of the executed movement with the right and left hand). These parameters were chosen since statistical tests comparing these variables between the high- and low-aptitude groups showed no statistical difference.

## Additional Information

**How to cite this article**: Marchesotti, S. *et al*. Quantifying the role of motor imagery in brain-machine interfaces. *Sci. Rep*. **6**, 24076; doi: 10.1038/srep24076 (2016).

## Supplementary Material

Supplementary Information

## References

[b1] Wolpaw J. R. & McFarland D. J. Control of a two-dimensional movement signal by a noninvasive brain-computer interface in humans. Proc. Natl. Acad. Sci. USA 101, 17849–54 (2004). 15585584 10.1073/pnas.0403504101 PMC535103

[b2] Pfurtscheller G. & Neuper C. Motor imagery and direct brain-computer communication. Proc. IEEE 89, 1123–1134 (2001).

[b3] Vidaurre C. & Blankertz B. Towards a cure for BCI illiteracy. Brain Topogr. 23, 194–8 (2010). 19946737 10.1007/s10548-009-0121-6 PMC2874052

[b4] Ahn M. & Chan S. Performance variation in motor imagery brain – computer interface: A brief review. J. Neurosci. Methods 243, 103–110 (2015). 25668430 10.1016/j.jneumeth.2015.01.033

[b5] Müller-Putz G. R., Scherer R., Brunner C., Leeb R. & Pfurtscheller G. Better than random? A closer look on BCI results. Int. Jouranl Bioelectromagn. 10, 52–55 (2008).

[b6] Halder S. . Prediction of brain-computer interface aptitude from individual brain structure. Front. Hum. Neurosci. 7, 105 (2013). 23565083 10.3389/fnhum.2013.00105 PMC3613602

[b7] Kasahara K., DaSalla C. S., Honda M. & Hanakawa T. Neuroanatomical correlates of brain–computer interface performance. Neuroimage 110, 95–100 (2015). 25659465 10.1016/j.neuroimage.2015.01.055

[b8] Halder S. . Neural mechanisms of brain-computer interface control. Neuroimage 55, 1779–90 (2011). 21256234 10.1016/j.neuroimage.2011.01.021

[b9] Blankertz B. . Neurophysiological predictor of SMR-based BCI performance. Neuroimage 51, 1303–1309 (2010). 20303409 10.1016/j.neuroimage.2010.03.022

[b10] Ahn M., Cho H., Ahn S. & Jun S. C. High theta and low alpha powers may be indicative of BCI-illiteracy in motor imagery. PLoS One 8, 1–11 (2013). 10.1371/journal.pone.0080886 PMC3838377 24278339

[b11] Bamdadian A., Guan C., Ang K. K. & Xu J. The predictive role of pre-cue EEG rhythms on MI-based BCI classification performance. J. Neurosci. Methods 235C, 138–144 (2014). 24979726 10.1016/j.jneumeth.2014.06.011

[b12] Hammer E. M. . Psychological predictors of SMR-BCI performance. Biol. Psychol. 89, 80–6 (2012). 21964375 10.1016/j.biopsycho.2011.09.006

[b13] Burde W. & Blankertz B. In Proc. 3rd Int. Brain–Computer Interface Work. Train. Course 2006 76–77 (Verlag der Technischen Universität Graz, 2006).

[b14] Randolph A. B., Jackson M. M. & Karmakar S. Individual Characteristics and Their Effect on Predicting Mu Rhythm Modulation. Int. J. Hum. Comput. Interact. 27, 24–37 (2010).

[b15] Hammer E. M., Kaufmann T., Kleih S. C., Blankertz B. & Kübler A. Visuo-motor coordination ability predicts performance with brain-computer interfaces controlled by modulation of sensorimotor rhythms (SMR). Front. Hum. Neurosci. 8, 1–9 (2014). 25147518 10.3389/fnhum.2014.00574 PMC4123785

[b16] Vuckovic A. & Osuagwu B. Using a motor imagery questionnaire to estimate the performance of a Brain-Computer Interface based on object oriented motor imagery. Clin. Neurophysiol. 124, 1586–95 (2013). 23535455 10.1016/j.clinph.2013.02.016

[b17] Neuper C., Scherer R., Wriessnegger S. & Pfurtscheller G. Motor imagery and action observation: modulation of sensorimotor brain rhythms during mental control of a brain-computer interface. Clin. Neurophysiol. 120, 239–47 (2009). 19121977 10.1016/j.clinph.2008.11.015

[b18] Jeannerod M. & Decety J. Mental motor imagery: a window into the representational stages of action. Curr. Opin. Neurobiol. 5, 727–732 (1995). 8805419 10.1016/0959-4388(95)80099-9

[b19] Mulder T. Motor imagery and action observation: cognitive tools for rehabilitation. J. Neural Transm. 114, 1265–78 (2007). 17579805 10.1007/s00702-007-0763-z PMC2797860

[b20] Sirigu A. . Congruent unilateral impairments for real and imagined hand movements. Neuroreport 6, 997–1001 (1995). 7632907 10.1097/00001756-199505090-00012

[b21] Collet C., Guillot A., Lebon F., Macintyre T. & Moran A. Measuring Motor Imagery Using Psychometric, Behavioral, and Psychophysiological Tools. Exerc. Sport Sci. Rev. 39, 85–92 (2011). 21206282 10.1097/JES.0b013e31820ac5e0

[b22] Papaxanthis C., Schieppati M., Gentili R. & Pozzo T. Imagined and actual arm movements have similar durations when performed under different conditions of direction and mass. Exp. brain Res. 143, 447–52 (2002). 11914790 10.1007/s00221-002-1012-1

[b23] Guillot A. . Brain activity during visual versus kinesthetic imagery: an fMRI study. Hum. Brain Mapp. 30, 2157–72 (2009). 18819106 10.1002/hbm.20658 PMC6870928

[b24] Lebon F., Byblow W. D., Collet C., Guillot A. & Stinear C. M. The modulation of motor cortex excitability during motor imagery depends on imagery quality. Eur. J. Neurosci. 35, 323–331 (2012). 22172012 10.1111/j.1460-9568.2011.07938.x

[b25] Bassolino M., Campanella M., Bove M., Pozzo T. & Fadiga L. Training the motor cortex by observing the actions of others during immobilization. Cereb. Cortex 24, 3268–76 (2014). 23897648 10.1093/cercor/bht190 PMC4224244

[b26] Gregg M., Hall C. & Butler A. The MIQ-RS: A Suitable Option for Examining Movement Imagery Ability. Evid. Based. Complement. Alternat. Med. 7, 249–57 (2010). 18955294 10.1093/ecam/nem170 PMC2862926

[b27] Zich C. . Real-time EEG feedback during simultaneous EEG–fMRI identifies the cortical signature of motor imagery. Neuroimage 114, 438–447 (2015). 25887263 10.1016/j.neuroimage.2015.04.020

[b28] Kübler A. . Brain-computer communication: self-regulation of slow cortical potentials for verbal communication. Arch. Phys. Med. Rehabil. 82, 1533–9 (2001). 11689972 10.1053/apmr.2001.26621

[b29] Guillot A. & Collet C. Duration of mentally simulated movement: a review. J. Mot. Behav. 37, 10–20 (2005). 15642689 10.3200/JMBR.37.1.10-20

[b30] McFarland D. J., Miner L. A., Vaughan T. M. & Wolpaw J. R. Mu and Beta Rhythm Topographies During Motor Imagery and Actual Movements. Brain Topogr. 12, 177–186 (2000). 10791681 10.1023/a:1023437823106

[b31] Guger C., Ramoser H. & Pfurtscheller G. Real-time EEG analysis with subject-specific spatial patterns for a brain-computer interface (BCI). IEEE Trans. Rehabil. Eng. 8, 447–56 (2000). 11204035 10.1109/86.895947

[b32] Combrisson E. & Jerbi K. Exceeding chance level by chance: The caveat of theoretical chance levels in brain signal classification and statistical assessment of decoding accuracy. J. Neurosci. Methods 250, 126–136 (2015). 25596422 10.1016/j.jneumeth.2015.01.010

[b33] Pfurtscheller G., Brunner C., Schlögl a. & Lopes da Silva F. H. Mu rhythm (de)synchronization and EEG single-trial classification of different motor imagery tasks. Neuroimage 31, 153–9 (2006). 16443377 10.1016/j.neuroimage.2005.12.003

[b34] Fourkas A. D., Ionta S. & Aglioti S. M. Influence of imagined posture and imagery modality on corticospinal excitability. Behav. Brain Res. 168, 190–6 (2006). 16313979 10.1016/j.bbr.2005.10.015

[b35] Lotze M. & Halsband U. Motor imagery. J. Physiol. Paris 99, 386–95 (2006). 16716573 10.1016/j.jphysparis.2006.03.012

[b36] Neuper C., Scherer R., Reiner M. & Pfurtscheller G. Imagery of motor actions: differential effects of kinesthetic and visual-motor mode of imagery in single-trial EEG. Brain Res. Cogn. Brain Res. 25, 668–77 (2005). 16236487 10.1016/j.cogbrainres.2005.08.014

[b37] Ietswaart M. . Mental practice with motor imagery in stroke recovery: Randomized controlled trial of efficacy. Brain 134, 1373–1386 (2011). 21515905 10.1093/brain/awr077 PMC3097892

[b38] Sirigu A. . The Mental Representation of Hand Movements After Parietal Cortex Damage. Science (80-.). 273, 1564–1568 (1996). 10.1126/science.273.5281.1564 8703221

[b39] Malouin F., Belleville S., Richards C. L., Desrosiers J. & Doyon J. Working memory and mental practice outcomes after stroke. Arch. Phys. Med. Rehabil. 85, 177–183 (2004). 14966700 10.1016/s0003-9993(03)00771-8

[b40] Liepert J., Greiner J., Nedelko V. & Dettmers C. Reduced Upper Limb Sensation Impairs Mental Chronometry for Motor Imagery After Stroke: Clinical and Electrophysiological Findings. Neurorehabil. Neural Repair 26, 470–478 (2012). 22247502 10.1177/1545968311425924

[b41] Johnson S. H., Sprehn G. & Saykin A. J. Intact motor imagery in chronic upper limb hemiplegics: evidence for activity-independent action representations. J. Cogn. Neurosci. 14, 841–52 (2002). 12191452 10.1162/089892902760191072

[b42] Prut Y. & Fetz E. E. Primate spinal interneurons show pre-movement instructed delay activity. Nature 401, 590–594 (1999). 10524626 10.1038/44145

[b43] Grosse-Wentrup M., Schölkopf B. & Hill J. Causal influence of gamma oscillations on the sensorimotor rhythm. Neuroimage 56, 837–842 (2011). 20451626 10.1016/j.neuroimage.2010.04.265

[b44] Bloom J. S. & Hynd G. W. The role of the corpus callosum in interhemispheric transfer of information: Excitation or inhibition? Neuropsychol. Rev. 15, 59–71 (2005). 16211466 10.1007/s11065-005-6252-y

[b45] Pfurtscheller G., Neuper C., Flotzinger D. & Pregenzer M. EEG-based discrimination between imagination of right and left hand movement. Electroencephalogr. Clin. Neurophysiol. 103, 642–51 (1997). 9546492 10.1016/s0013-4694(97)00080-1

[b46] Giovannelli F. . Modulation of interhemispheric inhibition by volitional motor activity: an ipsilateral silent period study. J. Physiol. 587, 5393–5410 (2009). 19770195 10.1113/jphysiol.2009.175885 PMC2793872

[b47] Liang N. . Effects of unilateral voluntary movement on motor imagery of the contralateral limb. Clin. Neurophysiol. 122, 550–557 (2011). 20800539 10.1016/j.clinph.2010.07.024

[b48] Gueugneau N. . Interhemispheric Inhibition during Mental Actions of Different Complexity. PLoS One 8, 1–8 (2013). 10.1371/journal.pone.0056973 PMC3581568 23451125

[b49] Perez M. A. & Cohen L. G. Mechanisms underlying functional changes in the primary motor cortex ipsilateral to an active hand. J. Neurosci. 28, 5631–40 (2008). 18509024 10.1523/JNEUROSCI.0093-08.2008 PMC2440822

[b50] Lebedev M. & Wise S. Oscillations in the premotor cortex: single-unit activity from awake, behaving monkeys. Exp. brain Res. 195–215 (2000). doi: 10.1007/s002210050022 10672473

[b51] Pesaran B., Pezaris J. S., Sahani M., Mitra P. P. & Andersen R. A. Temporal structure in neuronal activity during working memory in macaque parietal cortex. Nat. Neurosci. 5, 805–811 (2002). 12134152 10.1038/nn890

[b52] Wolpert D. M. & Flanagan J. R. Motor prediction. Curr. Biol. 11, R729–R732 (2001). 11566114 10.1016/s0960-9822(01)00432-8

[b53] Decety J. The neurophysiological basis of motor imagery. Behav. Brain Res. 77, 45–52 (1996). 8762158 10.1016/0166-4328(95)00225-1

[b54] Sirigu a. & Duhamel J. R. Motor and visual imagery as two complementary but neurally dissociable mental processes. J. Cogn. Neurosci. 13, 910–919 (2001). 11595094 10.1162/089892901753165827

[b55] Vargas C. D. . The influence of hand posture on corticospinal excitability during motor imagery: a transcranial magnetic stimulation study. Cereb. Cortex 14, 1200–6 (2004). 15142965 10.1093/cercor/bhh080

[b56] Sharma N., Pomeroy V. M. & Baron J. C. Motor imagery: A backdoor to the motor system after stroke? Stroke 37, 1941–1952 (2006). 16741183 10.1161/01.STR.0000226902.43357.fc

[b57] Jeannerod M. Mental imagery in the motor context. Neuropsychologia 33, 1419–1432 (1995). 8584178 10.1016/0028-3932(95)00073-c

[b58] Maclntyre T. & Moran A. In Neurophysiol. Found. Ment. Mot. Imag. (eds. Guillot A. & Collet C.) 227–244 (Oxford University Press, 2009).

[b59] Jeannerod M. Neural simulation of action: a unifying mechanism for motor cognition. Neuroimage 14, S103–9 (2001). 11373140 10.1006/nimg.2001.0832

[b60] Oldfield R. C. The assessment and analysis of handedness: the Edinburgh inventory. Neuropsychologia 9, 97–113 (1971). 5146491 10.1016/0028-3932(71)90067-4

[b61] Makeig S. Auditory event-related dynamics of the EEG spectrum and effects of exposure to tones. Electroencephalogr. Clin. Neurophysiol. 86, 283–293 (1993). 7682932 10.1016/0013-4694(93)90110-h

[b62] Fan R., Wang X. & Lin C. LIBLINEAR: A Library for Large Linear Classification. **9,** 1871–1874 (2014).

