# Supplementary material for: Quantifying the role of motor imagery in brain-machine interfaces
Source: Sci Rep. 2016 Apr 7;6:24076. doi: 10.1038/srep24076 (PMC4823701; doi:10.1038/srep24076)
Supplement: Supplementary Information [file srep24076-s1.doc]

**MANUSCRIPT TITLE:** Quantifying the role of motor imagery in brain-machine interfaces

**AUTHORS:** Silvia Marchesotti, Michela Bassolino, Andrea Serino, Hannes Bleuler, Olaf Blanke


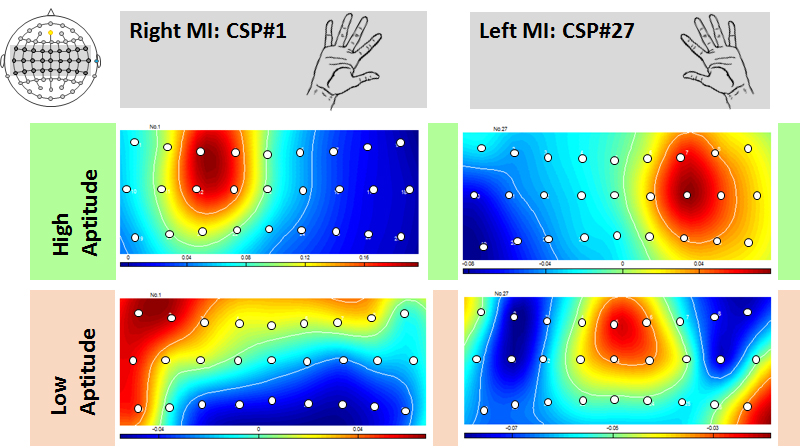


**Supplementary Figure 1.** **Common spatial patterns for the right and left motor imagery in two representative subjects**.

The upper panel shows the stereotypical pattern for both CSPs from a representative high- aptitude user indicating a stronger contribution in the classification by the electrodes placed in the hemisphere contralateral to the imaged hand. This pattern is not present in the CSPs from the representative low aptitude user (bottom row). White dots indicate the position of the BCI subset of 27-electrodes over the sensorimotor region, also highlighted in the configuration in the upper-left corner.
